# A pseudouridine synthase shapes tRNA structural dynamics through both catalysis and remodeling

**DOI:** 10.21203/rs.3.rs-6597546/v1

**Published:** 2025-05-23

**Authors:** Julia Widom, Emily Dennis, Nico Conoan Nieves, Abigail Vaaler, Madison Kadrmas, Maggie Barry, David Garcia

## Abstract

Transfer RNA has long served as an exemplar of a thermodynamically stable, structured RNA. Yet it undergoes significant structural changes upon binding and catalysis by diverse modification enzymes. We leveraged optical binding assays and single-molecule FRET to observe tRNA structural dynamics before and upon interaction with the conserved pseudouridine synthase Pus4/TruB. We show that unmodified and pseudouridylated tRNA similarly sample one open and two closed conformations, dynamically. Binding by Pus4 to unmodified tRNA populates additional conformational states, gradually approaching an ensemble that is adopted sooner by tRNA that was pseudouridylated prior to engaging Pus4. A catalytically incompetent mutant of Pus4 binds more slowly and remodels unmodified and pre-modified tRNAs into different conformational ensembles than wild-type enzyme. Thus, Pus4 both catalyzes a lasting chemical change on tRNA and remodels it over time, perhaps to advance subsequent steps of tRNA maturation.

### Introduction

Pseudouridine (Ψ) is the most abundant RNA chemical modification in nature. Position 55 in the T-loop is pseudouridylated in nearly all tRNAs, which is catalyzed by Pus4/TruB/TRUB1+TRUB2 (yeast/bacteria/human). This change from $U_{55}$ to $\Psi_{55}$ provides an additional hydrogen bond donor, increases base-stacking and helix rigidity, and promotes tertiary interactions between the T-arm and D-arm in the "elbow" region of a folded tRNA, contributing to the characteristic three-dimensional structure of tRNA[1–5]. In the budding yeast *Saccharomyces cerevisiae*, Pus4 (**Fig. 1a**) pseudouridylates 41 out of 42 cytosolic tRNA isoacceptors[6], and 23 out of 24 mitochondrial tRNA isoacceptors (Julia Reinsch, unpublished), and it does so with a high conversion efficiency of U to Ψ. In *E. coli*, all tRNAs are known to be pseudouridylated by TruB[8]. Therefore, the majority of tRNA molecules examined thus far are bound and modified by Pus4/TruB during at least one point of their biogenesis/maturation, implicating this enzyme as a benchmark for understanding how tRNA structure and dynamics are affected by an enzyme that chemically modifies it.

Previous studies have identified various RNA sequences and/or structures important for binding and modification by various pseudouridine synthase enzymes[9–25], including Pus4/TruB[26–28]. Yet binding by these synthase enzymes can remodel RNAs in ways distinct from the lasting chemical change that they catalyze. An early study on bacterial TruB noted that growth phenotypes associated with its absence could not be explained by its catalytic activity alone[29]. Subsequently, a second role was more clearly established: that it aids tRNA folding and functions as a tRNA chaperone[30]. Other studies have also demonstrated RNA-chaperone-like roles for other tRNA modifying enzymes[31–34]. Thus, to exert their full function, tRNA modifying enzymes should, 1) bind tRNA, and 2) catalyze chemical modification of tRNA; while some may also, 3) remodel tRNA structure. These three components could each independently affect the dynamics of tRNA structure, or do so interdependently. We sought to clarify these models by addressing the following questions: 1) Are there differences between the structural ensembles of unmodified tRNA and pseudouridylated tRNA? 2) How does binding by Pus4 affect the distribution of tRNA structures over time? 3) Does the outcome depend on whether the tRNA is modified prior to engagement, modified by Pus4, or not modified at all?

To address these questions, we leveraged single-molecule Förster resonance energy transfer (smFRET) to track the conformational fluctuations of tRNA and biolayer interferometry (BLI) to monitor association of Pus4 to tRNA (**Fig. 1c**). We show that Pus4 binds rapidly to both unmodified ($U_{55}$) and pseudouridylated ($\Psi_{55}$, hereafter also referred to as "pre-modified") tRNAs, and subsequently navigates unmodified tRNA through a pathway of catalysis and remodeling lasting tens of minutes, that is bypassed by pre-modified tRNA. This pathway involves a temporary population of conformations not prevalent for either $U_{55}$ or $\Psi_{55}$ tRNA in the absence of enzyme. We also show that a mutant with impaired binding and catalytic activity (Pus4 R286K) still remodels $U_{55}$ and $\Psi_{55}$ tRNAs, but into different conformational ensembles than those promoted by Pus4 wild-type (WT). Thus our results show that a prototypical RNA-modifying enzyme shapes the chronology of tRNA structural dynamics through both its catalysis and remodeling.

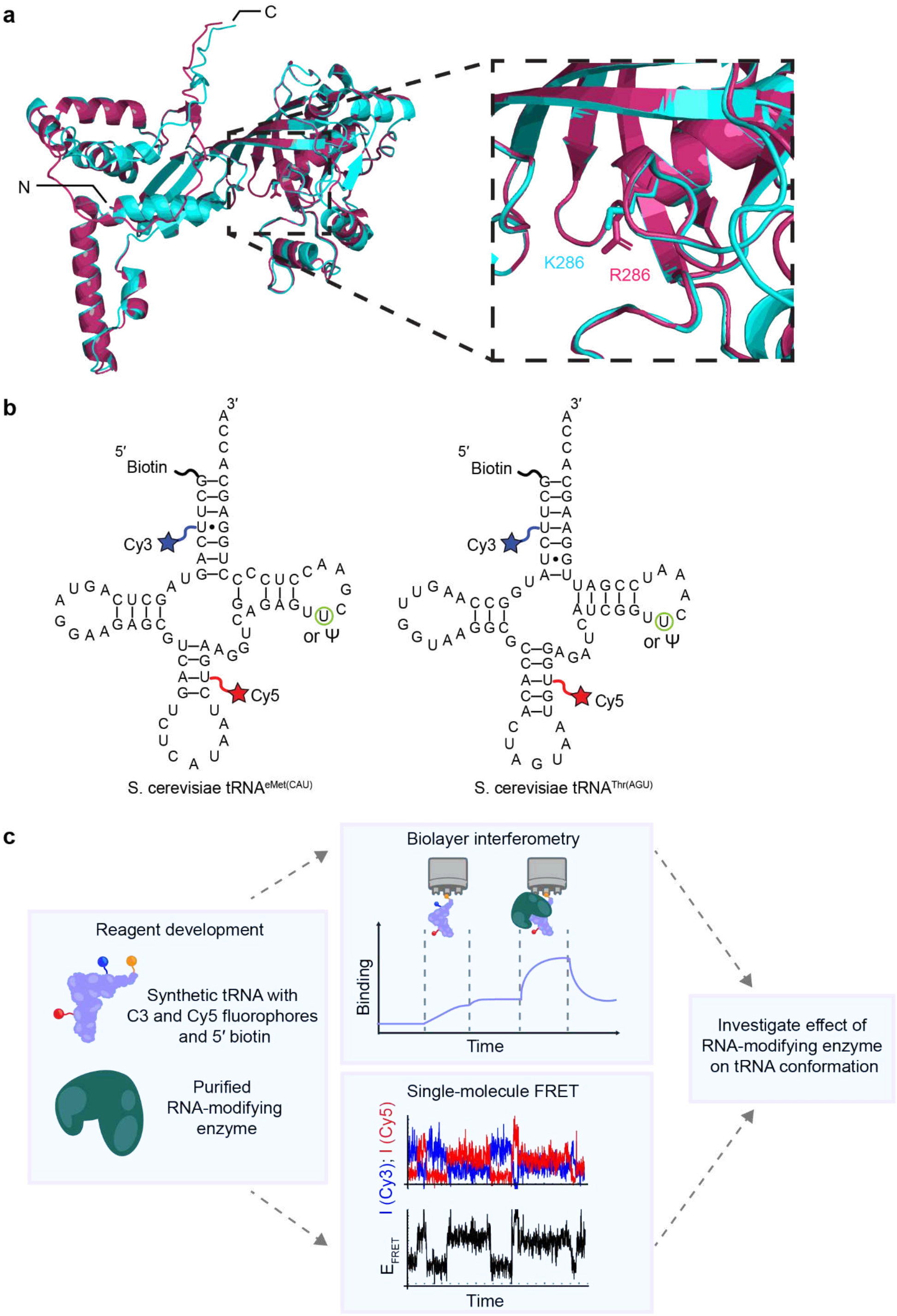

**Figure 1: Predicted Pus4 structure, tRNA sequences, and overall study approach.**
**a**, Structures of Pus4 WT (magenta) and Pus4 R286K (cyan) predicted with AlphaFold 3 and aligned using PyMol, with inset showing residue 286 in the active site. **b**, Predicted secondary structures of the tRNA sequences used in this study, including the positions of biotin, Cy3, Cy5 and pseudouridine modifications. **c**, Summary of experimental approach and workflow undertaken in this study.

### Results

#### Exogenously purified Pus4 pseudouridylates yeast tRNAs *in vitro*

After establishing a protocol to exogenously express and purify Pus4 protein (**Supp. Fig. 1a**), we tested its catalytic activity *in vitro* (**Supp. Fig. 2**). Using direct RNA sequencing (DRS), a method we previously established to investigate this modification *in vivo*[6], $\Psi_{55}$ was newly detected on nearly all tRNAs isolated from a *pus4*Δ yeast strain (**Supp. Table 1**) following incubation with an excess of purified Pus4 (**Supp. Fig. 2a**). Modification levels were qualitatively comparable to those previously observed in tRNA isolated from WT strains[6]. While some differences in pseudouridine levels were observed between the *in vivo* and *in vitro* data—most notably a lack of modification of tRNAs $Arg^{ACG}$ and $Leu^{CAA}$ *in vitro*—the data supported near-full catalytic activity of purified Pus4 on these cell-derived yeast tRNAs.

We previously identified a single point mutation to Pus4, converting arginine to lysine at position 286 (R286K; AlphaFold 3 structure prediction[35] in **Fig. 1a**), that impairs its pseudouridylation activity *in vivo*[6], and was based on a homologous mutant of TruB that also impairs catalysis without reducing tRNA binding *in vitro*[36]. Compared to Pus4 WT, incubation of R286K with yeast tRNAs *in vitro* produced almost no modification signal at $U_{55}$ across isoacceptors (**Supp. Fig. 2a**). Low levels of modification were observed on certain isoacceptors, however, the overall pattern resembled that of untreated tRNA isolated from *pus4*Δ strains. Thus Pus4 R286K is catalytically impaired *in vitro*.

#### Synthetic modified tRNAs enable binding and single molecule FRET studies

Having established recombinant Pus4 WT and R286K with desired enzymatic activities, we prepared synthetic tRNA for our biophysical studies of RNA binding by Pus4 (using BLI) and tRNA conformational fluctuations (using smFRET). We prepared synthetic $tRNA^{eMet(CAU)}$ and $tRNA^{Thr(AGU)}$ containing either U or Ψ at position 55, with modifications required for BLI and smFRET (**Fig. 1b**). Specifically, the 5′ ends were biotinylated for surface immobilization on a BLI probe/microscope slide, the FRET donor fluorophore Cy3 was placed in the acceptor stem, and the acceptor fluorophore Cy5 was placed in the anticodon stem, based on a previous design for human mt-$tRNA^{Lys}$ (ref)[37–40].

#### Wild-type Pus4 binds rapidly to tRNA

We utilized BLI to assess the binding specificity of Pus4 and the timescales of its association to and dissociation from both substrate and non-substrate RNAs. RNAs were immobilized on a streptavidin-coated probe that was then passivated to prevent non-specific adsorption of protein, exposed to buffers containing Pus4 protein (the "association phase"), and finally exposed to buffer lacking protein (the "dissociation phase") (**Fig. 2a, Supp. Fig. 3a**). A BLI "shift" is observed when material accumulates on the probe, such as protein binding to immobilized tRNA during the association phase. At a concentration of 200 nM protein, the association curves show rapid binding of Pus4 to $tRNA^{eMet(CAU)}$ and $tRNA^{Thr(AGU)}$, with the accumulation rate slowing after ~100 seconds (**Fig. 2b-d;** replicate data in **Supp. Fig. 4a-c**). This is followed by a period of slow continued accumulation of Pus4 onto tRNAs, observed at all protein concentrations tested. While this slowed accumulation plateaued after ~15 minutes

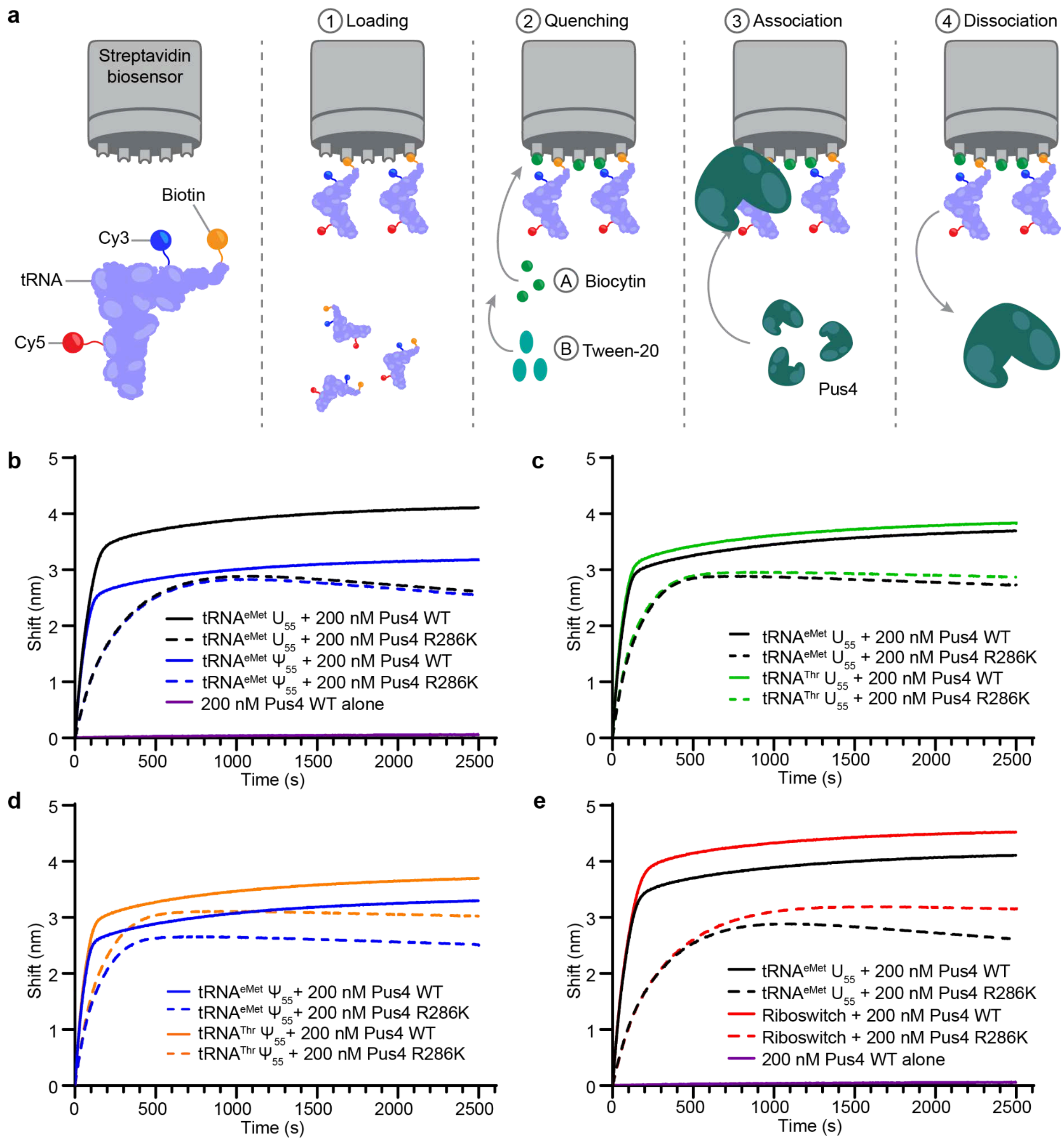

**Figure 2: Pus4 binding to tRNA measured using biolayer interferometry.**
**a**, Summary of BLI experimental design. Biotinylated and Cy3- and Cy5-labeled tRNAs are immobilized onto streptavidin-coated probes (1) that then undergo two quenching steps and baselining (2). The probe is then transferred into buffer with purified Pus4, where association of Pus4 with immobilized tRNAs occurs (3). The probe is transferred to buffer without protein, permitting dissociation (4). **b**, BLI traces showing the association phase of 200 nM Pus4 WT (solid) and Pus4 R286K (dotted) to both $U_{55}$ (black) and $\Psi_{55}$ (blue) $tRNA^{eMet(CAU)}$. Traces are aligned to start at a shift of 0 nm. **c**, Same as b, but with $U_{55}$ $tRNA^{eMet(CAU)}$ (black) and $U_{55}$ $tRNA^{Thr(AGU)}$ (green). **d**, Same as b, but with $\Psi_{55}$ $tRNA^{eMet(CAU)}$ (blue) and $\Psi_{55}$ $tRNA^{Thr(AGU)}$ (orange). Data collected in the same experiment as c. **e**, Same as b, but with $U_{55}$ $tRNA^{eMet(CAU)}$ (black) and $NAD^{+}$ riboswitch (red). $tRNA^{eMet(CAU)}$ data are the same as is shown in panel b.

when Pus4 was present at our highest tested concentration (800 nM), it failed to plateau even after 45 minutes for Pus4 at 50 or 200 nM concentrations (**Fig. 3a,b**). The BLI shift at 200 nM Pus4 eventually converged with the data for 800 nM, suggesting that complete binding occupancy was achieved in this concentration range. We confirmed this using non-denaturing gel electrophoresis as an orthogonal tRNA binding assay, which showed that in free solution, *all* $tRNA^{eMet(CAU)}$ molecules were bound after 30-minute incubation with Pus4 at concentrations of 200 nM or higher (**Supp. Fig. 5**). Combined with the prior observation that both $tRNA^{eMet(CAU)}$ and $tRNA^{Thr(AGU)}$ were robustly modified within two minutes during the *in vitro* modification experiment (in which Pus4 was present at 1 μM) (**Supp. Fig. 2**), we presumed that the majority of the immobilized tRNAs were pseudouridylated in the early period of this association phase once engaged with Pus4. The slow, steadily increasing second phase of the association curve may represent 1) gradual "trapping" of tRNA or Pus4 molecules that began the association phase in conformations not amenable to binding, or 2) a modest level of Pus4 multimerization such that the stoichiometry of Pus4:tRNA exceeded 1:1 on some of the immobilized tRNA molecules (the latter is supported by the shift of Pus4:tRNA bands to the wells for the highest Pus4 concentrations in **Supp. Fig. 5**).

We analyzed the association rates quantitatively by modeling the initial binding of Pus4 as a reversible process occurring under pseudo-first order conditions (details in **Methods**). Bi-exponential fits of the complete association curves and mono-exponential fits of the rapid phase (**Supp. Fig. 6**) both revealed that the on-rate ($k_{on}$) was slightly faster for $tRNA^{Thr(AGU)}$ ($1.36*10^5$ $M^{-1}s^{-1}$ from the bi-exponential fit) than for $tRNA^{eMet(CAU)}$ ($1.00*10^5$ $M^{-1}s^{-1}$) (**Fig. 3c,d**). These on-rates are comparable to ones previously observed for yeast Pus1 binding to $tRNA^{Phe}$ and $tRNA^{Val}$ ($4\text{-}6*10^5$ $M^{-1}s^{-1}$)[41]. These fits could in principle have been used to obtain $k_{off}$, however the off-rate during the initial phase of Pus4 accumulation was indistinguishable from zero. When the BLI probe was transferred into a solution lacking protein, an initial fast phase of dissociation was observed, but even after 45 minutes, less than 10% of the shift observed in the association phase was reversed (**Supp. Fig. 3b-d**). This suggests that Pus4 WT binds all RNAs investigated with high affinity and dissociates very slowly.

**Pus4 also binds robustly to pre-modified tRNA and non-substrate RNA**

To test whether $\Psi_{55}$ altered the binding of Pus4 to tRNA, we tested synthetic tRNAs that had pseudouridine present at position 55 using BLI. Although the BLI curve of $\Psi_{55}$ $tRNA^{eMet(CAU)}$ exhibited a smaller maximum shift than that of $U_{55}$ (**Fig. 3a**), non-denaturing gel electrophoresis indicated that this still corresponded to complete binding occupancy at Pus4 concentrations at or above 200 nM (**Supp. Fig. 5**). Furthermore, the overall association and dissociation profile was similar between the unmodified and pre-modified variants of both tRNAs, and $k_{on}$ values were unchanged or higher with $\Psi_{55}$ tRNA ($1.12*10^5$ $M^{-1}s^{-1}$ for $\Psi_{55}$ $tRNA^{eMet(CAU)}$ and $1.36*10^5$ $M^{-1}s^{-1}$ for $\Psi_{55}$ $tRNA^{Thr(AGU)}$), indicating weak discrimination between binding substrate and product. It is worth noting that previous studies have shown pseudouridine synthases can be promiscuous binders of RNA[9,11–13], consistent with their roles of modifying multiple sequences and structures in various classes and sizes of RNA. For example, Pus1 has been found to have broad affinity for RNA, even those lacking defining tRNA structural features, as well as tight binding to catalytic products (e.g., $K_D$ = 14 nM for pseudouridylated $tRNA^{Val}$)[42]. In addition to

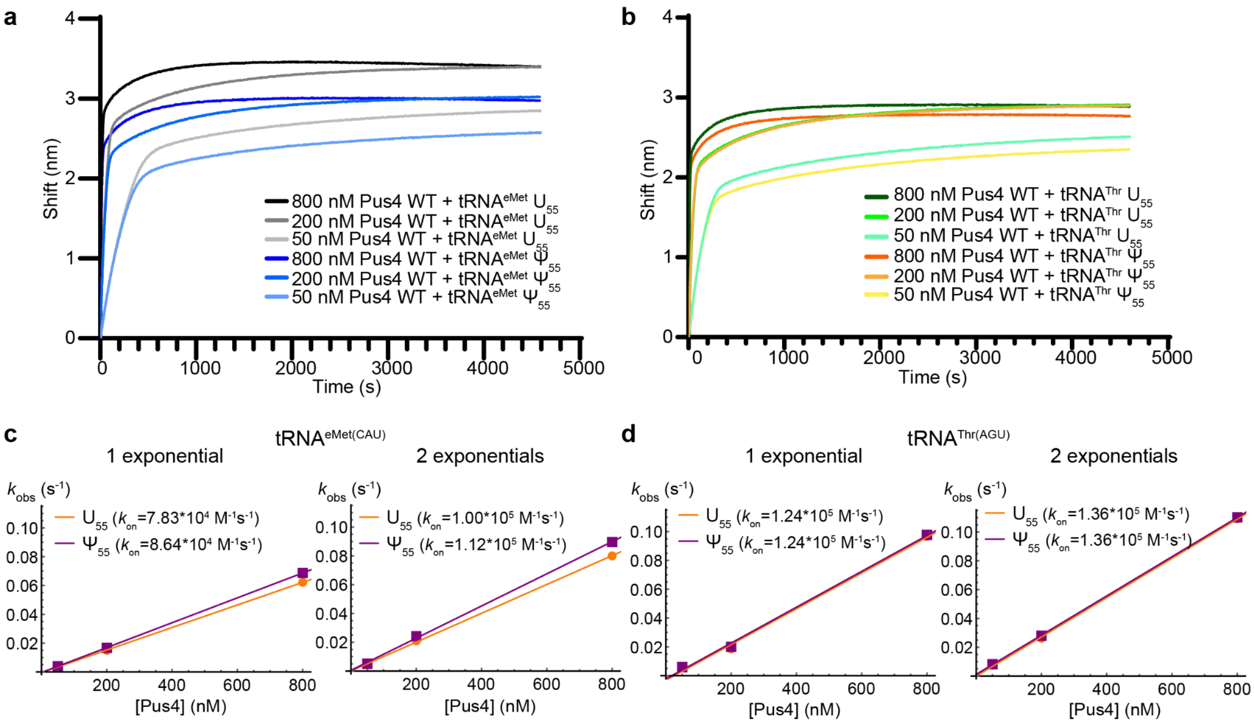

**Figure 3: Pus4 binds tRNAs with a high $k_{on}$, but a low $k_{off}$, with similar affinities for unmodified and pre-modified tRNAs.**
**a**, BLI traces showing the association phase of 50 nM, 200 nM, and 800 nM Pus4 WT to $U_{55}$ (black) and $\Psi_{55}$ (blue) $tRNA^{eMet(CAU)}$. **b**, BLI traces showing the association phase of 50 nM, 200 nM, and 800 nM Pus4 WT to $U_{55}$ (green) and $\Psi_{55}$ (orange) $tRNA^{Thr(AGU)}$. **c**,**d,** Linear fits to the observed association rate at three Pus4 concentrations. Left columns: $k_{obs}$ determined from a single exponential fit to the time range of the association phase during which ~75% of the BLI shift occurs. Right columns: $k_{obs}$ determined from a double exponential fit to the entire association phase. **c**, $U_{55}$ (orange) and $\Psi_{55}$ (purple) $tRNA^{eMet(CAU)}$. **d**, $U_{55}$ (orange) and $\Psi_{55}$ (purple) $tRNA^{Thr(AGU).}$

tRNA, Pus4 modifies mRNA[21,43,44] and rRNA[45], *in vivo*. To better contextualize our BLI results, we also performed experiments with an RNA that is not a natural substrate of Pus4 but is similarly sized to tRNA: the bacterial class II $NAD^+$ riboswitch, a 61 nt RNA that adopts a pseudoknot structural motif to regulate translation initiation[46,47]. As we observed for tRNA, Pus4 rapidly associated with the riboswitch, followed by slow continued accumulation and slow dissociation (**Fig. 2e, Supp. Fig. 3e**). We note the possibility that *in vivo*, re-binding of tRNA by Pus4 may be repressed by $m^1A_{58}$, that depends on $\Psi_{55}$ (ref)[6,7]. *In vivo*, most tRNA modifications are capable of being added to tRNAs lacking $\Psi_{55}$ (ref)[6]; however, our *in vitro* data also demonstrated that these modifications did not generally inhibit exogenous Pus4's ability to pseudouridylate them (**Supp. Fig. 2**).

**A catalytically-inactive mutant of Pus4 has impaired binding to tRNA**

To determine how Pus4's catalytic function influences its binding to tRNA, we performed BLI experiments with Pus4 R286K. This mutant showed a reduced rate of initial association to tRNA as well as a lower maximum occupancy (**Fig. 2b-d**). Pus4 R286K had impaired binding to the riboswitch as well, suggesting broad impairment of its RNA binding capability (**Fig. 2e**). After around 800 seconds (~13 minutes) of the association phase at 200 nM Pus4, the catalytically inactive mutant began a slow but steady dissociation from substrate (**Fig. 2b**). This behavior was identical for unmodified and pre-modified tRNA sequences of both isoacceptors, though it was not observed for the riboswitch **(Fig. 2b-e)**. We ruled out RNase contamination as a possible explanation for the decreasing association signal in these samples (**Supp. Fig. 1b**). It is possible this decrease in signal results from a population of tRNA molecules that acquire a structure bound more weakly by this mutant, although this is difficult to disentangle in a bulk measurement. Together, these data suggest that the active site residue R286 is required for catalytic activity and rapid association with tRNA, as well as promoting continued engagement or re-engagement with tRNA molecules.

**tRNA undergoes dynamic fluctuations between three predominant conformational states**

We utilized smFRET to investigate how tRNA conformations are affected by Pus4 binding and pseudouridylation. The biotinylated- and fluorophore-labeled $tRNA^{eMet(CAU)}$ constructs used for BLI were immobilized on microscope slides, and fluorescence from single tRNA molecules was recorded using prism-based total internal reflection fluorescence (TIRF) microscopy. We recorded short movies (~2 minutes) sequentially across different regions of the slide of tRNA alone, as well as tRNA during up to 30 minutes of incubation with 200 nM Pus4 protein. Cy3 and Cy5 fluorescence intensity traces were extracted from individual molecules, and data from at least two separate experiments were combined to achieve 200 or more individual molecules for each condition. In order to observe evolution of the conformational landscape over tens of minutes, for samples incubated with protein, the data were combined into discrete time bins of 2–6 minutes, 7–9 minutes, 10–15 minutes and 30–34 minutes after introduction of Pus4 (see **Methods** for an explanation of bins).

In the absence of protein, both $U_{55}$ and $\Psi_{55}$ tRNAs dynamically sample several different conformational states. Three are visible upon inspection of individual traces (**Fig. 4a,b**) and in histograms compiled from the first 50 frames of N > 200 traces (**Fig. 4c,d**). A low-FRET efficiency feature (LF)—representing a conformation in which the fluorophores are most separated from each other relative to other states—is centered around $E_{FRET} = 0.30$ for $U_{55}$, and 0.22 for $\Psi_{55}$ tRNA. An LF feature could arise from molecules that are either less structured or that adopt a structure such as an extended helix that enforces separation of the fluorophores. Two high-FRET features (HF1 and HF2)—representing conformations in which the fluorophores are closer to each other relative to LF—were observed for both tRNAs and are centered around $E_{FRET} = 0.77$ and 0.98 for HF1 and HF2, respectively (**Fig. 4c,d**). The LF and HF1/HF2 states we observed may approximate the U (unfolded) and C (cloverleaf-based-L-shape) conformations, respectively, that were previously identified through smFRET analysis of the human $mt\text{-}tRNA^{Lys}$ (ref)[37]. We hypothesize that the HF1 and HF2 states we have defined here share the overall C secondary structure, yet differ in certain tertiary contacts. In summary, we observed three

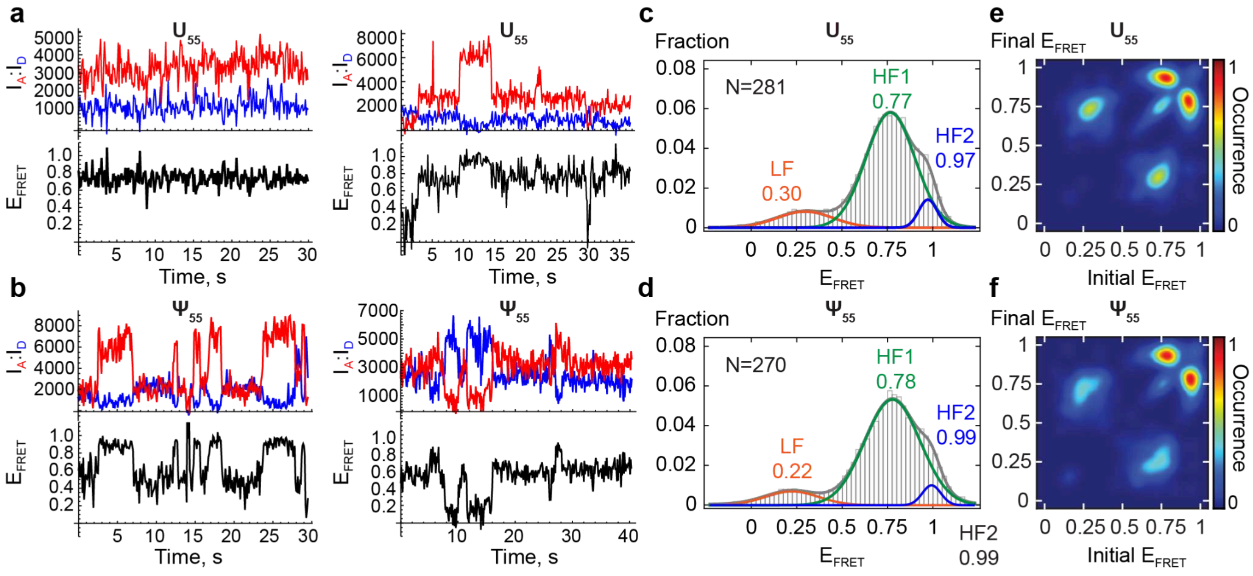

**Figure 4: smFRET histograms and TODPs for $U_{55}$ and $\Psi_{55}$ tRNAs.**
**a**, Representative donor (blue), acceptor (red) and $E_{FRET}$ (black) traces of $U_{55}$ $tRNA^{eMet(CAU)}$ in the absence of Pus4. **b**, Same as a, but for $\Psi_{55}$ tRNA. **c**, Histogram of the first 50 frames of N=281 traces for $U_{55}$ tRNA. A 3-Gaussian fit is overlaid with the peak centers labeled. **d**, Same as c, but for $\Psi_{55}$ tRNA (N=270). **e**, TODP for $U_{55}$ tRNA, where the intensity of the heatmap at position (X,Y) quantifies the fraction of molecules that transition from $E_{FRET}$ = X to $E_{FRET}$ = Y at least once during the measurement. **f**, Same as e, but for $\Psi_{55}$ tRNA.

predominant conformational states in the absence of protein, though this behavior was highly similar for $U_{55}$ and $\Psi_{55}$ tRNAs.

We analyzed the dynamics of $U_{55}$ and $\Psi_{55}$ $tRNA^{eMet(CAU)}$ by performing Hidden Markov Modeling (HMM)[48] using a 3-state model that included the LF, HF1, and HF2 states. The pathways of transitions among these three states were visualized using transition occupancy density plots (TODPs), which show the prevalence of dynamic or static behavior across hundreds of molecules within each condition (**Fig. 4e,f**). A "feature" on the diagonal axis of a TODP (i.e. lower left corner to upper right corner) indicates a population of molecules that remain in one state throughout their measurement, while an off-diagonal feature at position (X,Y) indicates a population of molecules that transition from states with $E_{FRET}$ = X (initial) to $E_{FRET}$ = Y (final) at some point during the measurement. This analysis showed that a minority of molecules (~5% for both $U_{55}$ and $\Psi_{55}$) remained in the HF1 or HF2 state for longer than 30 seconds, while the majority of molecules exhibited dynamic behavior involving fluctuations between conformations. This is consistent with previous bulk[49,50] and smFRET[40] studies that showed that tRNAs can interconvert between multiple secondary and tertiary structures at physiological salt and temperature. In both $U_{55}$ and $\Psi_{55}$ tRNAs, bright off-diagonal features in the TODPs indicate that tRNAs readily and reversibly interconverted between the LF and HF1 states and between the

HF1 and HF2 states (**Fig. 4e,f**). In contrast, almost no intensity was observed at the locations corresponding to transitions between the LF and HF2 states. We analyzed the rates of these transitions using cross-correlation analysis (details in **Methods**), which identifies how rapidly Cy3 and Cy5 intensities become uncorrelated with one another within a collection of smFRET traces. The cross-correlation curves of $U_{55}$ and $\Psi_{55}$ tRNAs were both well-fit with a mono-exponential decay with a time constant of ~1 second (**Supp. Fig. 7**), suggesting that interconversion between LF and HF1 and between HF1 and HF2 both occur on this time-scale. In summary, our analysis revealed that $U_{55}$ and $\Psi_{55}$ tRNAs can undergo frequent conformational changes involving substantial changes in their 3-dimensional architecture.

**Pus4 alters the conformational states and their transition dynamics in tRNA**

After Pus4 was added to $U_{55}$ tRNA, a rapid increase in conformational complexity was observed, indicating that the protein changed the free energy landscape of tRNA molecules (**Fig. 5a**). The earliest time window probed by smFRET, 2–6 minutes, begins when the initial fast phase of Pus4 association observed by BLI is nearly complete, and continues into the slow accumulation phase (**Fig. 2b-d**). Inspection of individual traces recorded during this time window revealed a new population of mid-FRET (MF) states with $E_{FRET}$ between 0.4–0.6 (**Supp. Fig. 8)**, which were not among the predominant states in $U_{55}$ tRNA alone. Consistent with this observation, transitions between the LF and HF1 states were less clearly resolved in the TODPs, with a cluster of off-diagonal features representing discrete "microstates" with $E_{FRET}$ between 0.5–0.8 interconverting with microstates with $E_{FRET}$ between 0.2–0.5. As observed in tRNA alone, transitions between HF1 and HF2 remained well resolved after addition of Pus4 (**Fig. 5a-d**), although a subtle shift in $E_{HF1}$ from 0.77 in $U_{55}$ alone, to 0.80 after 2–6 minutes of incubation with Pus4, may indicate changes to specific tertiary contacts previously held (**Supp. Fig. 9a**). After 7–9 minutes of incubation, less sampling of MF states was observed, and the peak $E_{FRET}$ value for HF1 returned to 0.77. By 10–15 minutes after addition of Pus4, $U_{55}$ tRNA mostly ceased sampling MF states in lieu of well-resolved HF1–LF or HF1–HF2 transitions, resembling behavior observed in tRNA-only data. After 30-34 minutes (at which point all tRNA molecules are bound by Pus4, **Supp. Fig. 5**), a significant increase in the occupancy of HF2 was observed relative to earlier time points (**Supp. Fig. 9a**). Therefore, within the first 15 minutes after addition of Pus4, it altered tRNA conformation by promoting temporary sampling of MF states that were nearly absent without protein. We hypothesize that these MF states involve remodeling of the T-loop in order to facilitate pseudouridylation, as observed in crystallography data of the bacterial homolog TruB with tRNA[51,52]. While our approach precluded real-time, simultaneous measurement of pseudouridylation of tRNA immobilized on microscope slides, our *in vitro* pseudouridylation assay showed that the majority of tRNAs were pseudouridylated by Pus4 WT within 2 minutes (**Supp. Fig. 2a**). Therefore, results over the course of these smFRET experiments reflect the enduring influence of catalysis on the trajectory of the tRNA conformations sampled.

To help isolate the influence of Pus4 binding and remodeling of tRNA from its pseudouridylation activity, we performed smFRET experiments with the pre-modified $\Psi_{55}$ $tRNA^{eMet(CAU)}$ in the presence of Pus4. In contrast to the dramatic changes observed in $U_{55}$ tRNA shortly after addition of Pus4, $\Psi_{55}$ tRNA's smFRET traces resolved into sharp yet dynamic LF, HF1, and HF2

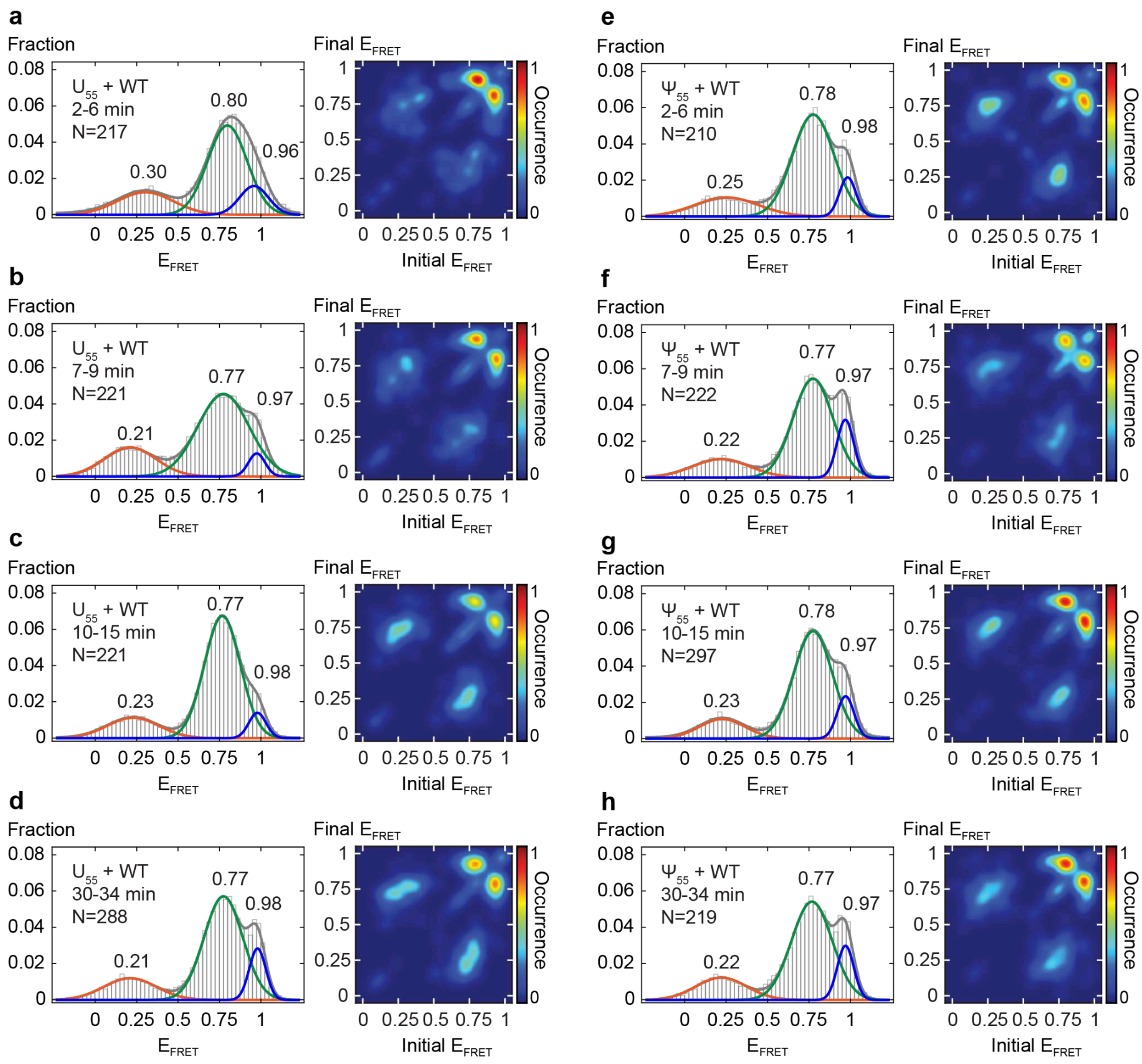

**Figure 5: smFRET histograms and TODPs for $U_{55}$ and $\Psi_{55}$ tRNAs with Pus4 WT.**
**a-d** Histograms of the first 50 frames of N>200 traces and TODPs for $U_{55}$ tRNA at various time points following addition of Pus4 WT **a**, 2-6 minutes **b**, 7-9 minutes **c**, 10-15 minutes **d**, 30-34 minutes **e-h**, Same as a-d, but for $\Psi_{55}$ tRNA.

states as early as the 2–6 minute window (**Fig. 5e-h**). Pus4 slightly increased the population of the LF state of $\Psi_{55}$ tRNA rapidly after binding (also seen with $U_{55}$) (**Supp. Fig. 9b**), but it did not promote as much sampling of MF states, which we propose are repressed by the presence of $\Psi_{55}$. Furthermore, the initial shift of HF1 to higher $E_{FRET}$ exhibited by $U_{55}$ tRNA was not observed in $\Psi_{55}$ tRNA. We hypothesize that $\Psi_{55}$ tRNA thus bypassed some conformational changes that $U_{55}$ tRNA transitioned through during its catalysis and subsequent remodeling by Pus4. In agreement with this model, the conformational landscape of $U_{55}$ tRNA evolved toward the more steady conformational landscape of $\Psi_{55}$, but required 30 minutes to attain the distribution of structures that $\Psi_{55}$ attained in only a few minutes (**Fig. 5, Supp. Fig. 9e,f**).

#### Catalytically-inactive Pus4 alters the tRNA remodeling pathway

While the Pus4 R286K mutant had impaired binding and catalysis, we found that it still promoted structural remodeling. It initially influenced $U_{55}$ and $\Psi_{55}$ tRNA similarly (**Fig. 6a,e**),

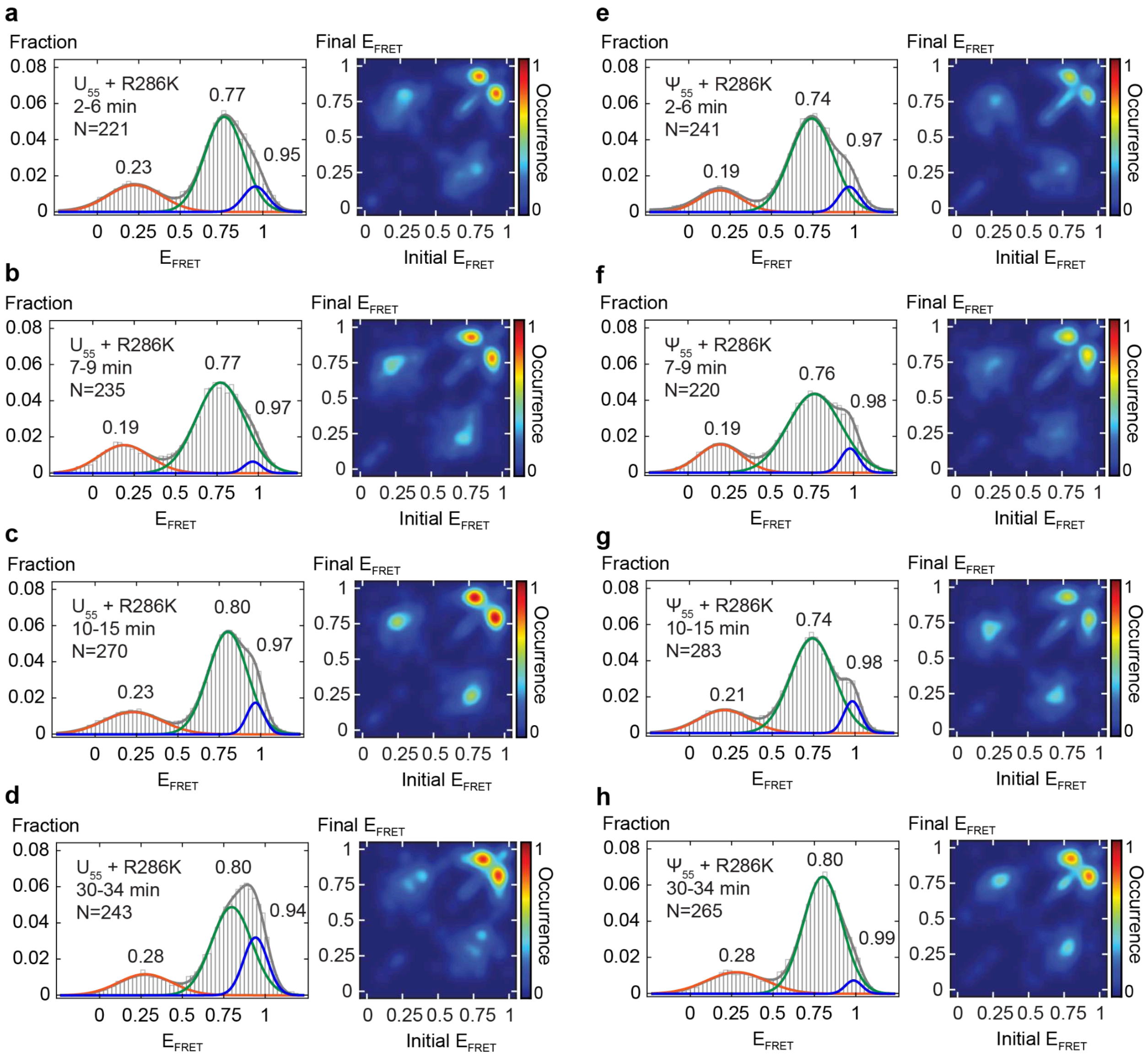

**Figure 6: smFRET histograms and TODPs for $U_{55}$ and $\Psi_{55}$ tRNAs with Pus4 R286K.**
**a-d** Histograms of the first 50 frames of N>200 traces and TODPs for $U_{55}$ tRNA at various time points following addition of Pus4 R286K **a**, 2-6 minutes **b**, 7-9 minutes **c**, 10-15 minutes **d**, 30-34 minutes **e-h**, Same as a-d, but for $\Psi_{55}$ tRNA.

unlike Pus4 WT which remodeled the two differently at early time points (**Fig. 5a,e**). During the 2–6 minute time period with Pus4 R286K, the population of the LF state increased and tRNA sampled a diverse array of MF conformations (**Fig. 6a,e**), despite only being bound at ⅓–⅔ of its peak protein occupancy (**Fig. 2b**). The distribution of MF states sampled was less broad than observed for $U_{55}$ + Pus4 WT at early time points (**Fig. 5a**), as indicated by the persistence of a prominent feature (rather than a cluster of features) at the locations corresponding to LF ↔ HF1 transitions in their TODPs (**Fig. 6a,e**). This suggests that the mutant is less effective at facilitating the exploration of different structures. In contrast to their similarity at early times, the conformational landscapes of $U_{55}$ and $\Psi_{55}$ tRNA diverged significantly by the 30-34 minute window **(Fig. 6d,h, Supp. Fig. S9e–h**). With protein binding occupancy still close to its peak (**Fig. 2b)**, $U_{55}$ tRNA maintained transitions between HF1 and both LF and MF states, as was observed at early time points for Pus4 WT. In contrast, $\Psi_{55}$ tRNA exhibited much less sampling of MF states and a smaller HF2 population, compared to $U_{55}$ tRNA (**Fig. 6d,h**). Thus, despite the deficiency of Pus4 R286K in discriminating between $U_{55}$ and $\Psi_{55}$ tRNAs immediately after binding, it ultimately remodels them into different structural ensembles. These results suggest that Pus4 R286K can still remodel tRNA, with the presence or absence of $\Psi_{55}$ impacting the outcome.

### Discussion

For many decades since its three-dimensional structure was first elucidated[53], tRNA has been presented as an archetype of structured non-coding RNA. Yet every tRNA undergoes many processing steps during biogenesis—including trimming, tailing and addition of multiple chemical modifications—necessitating many conformational changes. In this study, we combined biophysical and biochemical approaches to document the dynamics of these conformational changes using a yeast tRNA and a ubiquitous pseudouridine synthase as a model. Our data demonstrate how Pus4 impacts tRNA conformation through its binding, promotion of conformations compatible with catalysis, and resolution toward a more sharply defined ensemble of conformations. The data show this final step occurring over a period of minutes if catalysis is bypassed (i.e. Pus4 WT+pre-modified tRNA) or *tens* of minutes if catalysis occurs or is precluded through interaction with catalytically-incompetent enzyme. Pus4 R286K influenced the conformational states explored by the tRNA differently than Pus4 WT did, thus demonstrating the impact of a single active site residue on the ability of Pus4 to bind to, chemically modify *and* remodel tRNA.

We find that tRNA maintains the fundamental conformational landscape of LF, HF1 and HF2 states throughout its interactions with Pus4 and independently of its modification status, with MF states being populated only under certain circumstances. We consistently observe a higher HF population in the yeast $tRNA^{eMet(CAU)}$ profiled in this study than previously observed by smFRET in human mt-$tRNA^{Lys}$ (ref)[37–40], which we propose can be reconciled by predicted free energies. The predicted cloverleaf secondary structure of the tRNA that we investigated has a predicted $\Delta G_{folding}$ = -18.9 kcal/mol, versus -16.1 kcal/mol for human mt-$tRNA^{Lys}$ studied in Voigts-Hoffman *et al.*, 2007 (details of free energy calculations in **Methods**) (**Supp. Fig. 10**). mt-$tRNA^{Lys}$

populated a mid-FRET state with $E_{FRET}$ ~0.4 that interconverted with C on a time-scale of hundreds of milliseconds, which the authors assigned to an extended hairpin secondary structure (E)[40]. Interconversion between C- and E-type structures was also observed in a different tRNA, mt-$tRNA^{Leu}$ (ref)[50], using biochemical methods. We identified a plausible secondary structure of $tRNA^{eMet(CAU)}$ that mimics the E structure but, at $\Delta G$ = -12.9 kcal/mol, it is 6 kcal/mol less stable than this tRNA's predicted C structure (**Supp. Fig. 10a,b**), potentially explaining its low prevalence. In contrast, the E structure proposed in Voigts-Hoffman *et al.*, 2007 is predicted to have a free energy of -15.3 kcal/mol, within 1 kcal/mol to that of the C structure (**Supp. Fig. 10c,d**). Differences between the relative stabilities of E (non-native) and C structures have been previously shown to influence tRNA biogenesis and neurodegeneration[54]. Once Pus4 was introduced, we observed a rapid increase in MF conformations, though our smFRET approach does not distinguish whether these resemble the aforementioned E structure, or originate from altered tertiary contacts. Future studies of tRNA models with alternate placement of FRET pairs may enable delineation of these structures.

The resolution of the C structure of $tRNA^{eMet(CAU)}$ into two distinct states (HF1 and HF2) is another noteworthy observation in our study, and is consistent with prior steady-state and kinetic measurements that identified multiple interconvertible tertiary structures under conditions generally favoring the C secondary structure[49,55]. Splitting of the HF feature into two distinct states was not observed for human mt-$tRNA^{Lys}$ under any condition reported; instead its C conformation consistently peaked around $E_{FRET}$ ~0.8 over a variety of ionic conditions and chemical modification states[37–40]. It is possible that the low thermodynamic stability of the C structure of mt-$tRNA^{Lys}$ precludes multiple stable tertiary structures from being resolved within the time resolution of the smFRET measurements reported for it.

We summarize our results with a model for the conformational dynamics of yeast $tRNA^{eMet(CAU)}$, and how they are influenced by pseudouridylation and interaction with Pus4 (**Fig. 7**). We propose that each instance of Pus4 engaging with tRNA follows a three-to-four-step process: 1) Initial binding of Pus4 occurs. 2) Pus4 "tests" whether $\Psi_{55}$ is already present. 3) If $\Psi_{55}$ is not present, Pus4 can convert $U_{55}$ to $\Psi_{55}$. 4) Pus4 remodels tRNA into its final distribution of structures. Pus4 WT effectively discriminates U from Ψ, bypassing catalysis for pre-modified tRNA and remodeling within minutes, as indicated by the rapid convergence of the structural ensemble when Pus4 has bound $\Psi_{55}$ tRNA. In checking for $\Psi_{55}$ prior to catalysis, Pus4 induces an additional population of intermediate (MF) states, including states that may promote catalysis, or an indiscriminate sampling of states similar to the catalytically active one (e.g. both tRNAs + Pus4 R286K). Overall our results demonstrate two important layers of influence on tRNA conformational dynamics: chemical catalysis and remodeling resulting from extended interactions with modification enzymes. Why Pus4 should exert such malleability toward tRNA conformational dynamics, even on already pseudouridylated tRNAs, remains an important question. Since Pus4 catalysis can strongly influence the addition of subsequent modifications[6], some added over many hours after pseudouridylation[7], we propose that part of the role of these enzymes is to make tRNA structure amenable to binding and modification by subsequent modification enzymes critical for tRNA function.

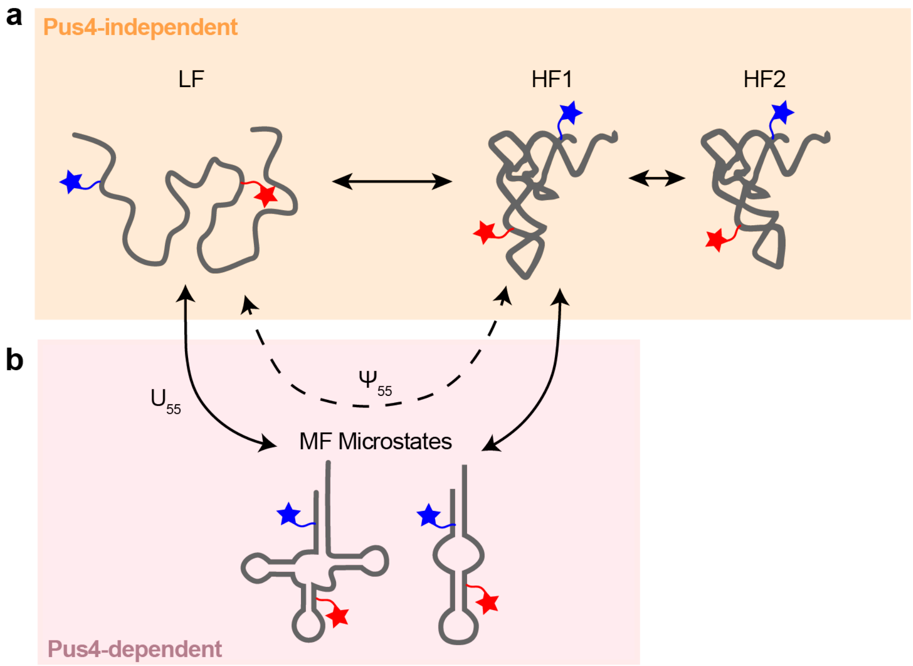

**Figure 7: Proposed model for the conformational dynamics of tRNA as influenced by Pus4**

**a**, tRNAs interconvert between multiple cloverleaf-based conformations (HF1, HF2) and an extended (LF) conformation, independent of pseudouridine modification. **b**, Addition of Pus4 adds a new population of intermediate (MF) states in $U_{55}$ tRNA that eventually resolve into LF and HF states after 30 or more minutes. This step is bypassed by $\Psi_{55}$ + Pus4 WT, which more quickly resolves to HF1 and HF2 states. Pus4 may enable additional secondary structures to be accessed in the MF states, such as an extended hairpin.

### Methods

#### Synthetic tRNA sequence design and preparation

Fluorophore placement was modeled after that used in Voigts-Hoffmann *et al.*, 2007[40]. The yeast $tRNA^{eMet(CAU)}$ and $tRNA^{Thr(AGU)}$ were chosen for several reasons: 1) both contain uridines at positions 4 and 41, which were the positions labeled with fluorophores and match those labeled in the prior work[40], 2) both have a minimum free energy structure as predicted by RNAFold[56,57], which closely resembles the tRNA cloverleaf secondary structure, theoretically reducing the propensity for the formation of non-canonical structures *in vitro*, and 3) prior evidence suggests both tRNAs are pseudouridylated by Pus4 efficiently *in vivo*[6] and our work herein demonstrated their pseudouridylation by Pus4 *in vitro*. The tRNAs were designed to contain Cy3 attached to the carbon-5 position of U4 and Cy5 attached to the carbon-5 position of U41, as well as biotinylation of the 5′-phosphate to enable attachment of the molecules to the microscope slide or probe for smFRET and BLI, respectively.

Full-length tRNA constructs were made by ligation of two RNA oligos (Dharmacon, HPLC-purified by the manufacturer) using a DNA splint (Integrated DNA Technologies, PAGE-purified by the manufacturer) (**Supp. Table 2**). Oligonucleotide concentrations were quantified using A260 measurements on a Nanodrop spectrophotometer and extinction coefficients provided by the manufacturer. Ligation was performed using 2 nmol of each of three oligos in a final volume of 67.5 µL containing 7.5 µL hybridization buffer (150 mM HEPES pH 7.5 and 500 mM KOAc). This solution was heated for 2 minutes at 90°C and allowed to cool at room temperature for 30 seconds before 10X T4 RNA ligase 2 buffer (NEB) was added to a final concentration of 1X. The mixture was then cooled at room temperature for an additional 10 minutes, at which time additional ligase buffer, ATP, water, and T4 RNA ligase 2 (MC Labs) were added to achieve a reaction volume of 100 µL containing 0.4 mM ATP, 1X RNA ligase 2 buffer, and 10 µL (100 units) T4 RNA ligase 2. The ligation mixture was incubated at room temperature for 3 hours. Following this, 8 µL (16 units) DNase I (NEB) was added along with 10X DNase I buffer to bring the final concentration of that buffer to 1X. This mixture was incubated at 37°C for 15 minutes to digest the DNA splint.

The ligation mixture was heated for 2 minutes at 90°C to inactivate the enzyme and unfold the tRNA, and then run on a 12% polyacrylamide, 8 M urea denaturing gel. Gels were imaged using an Amersham Typhoon (GE) and further visualized using a Dark Reader transilluminator (Clare Chemical Research). The 76 nt band corresponding to the full length tRNA was extracted by electro-elution, and the product was isolated by ethanol precipitation.

#### Protein expression and purification

Starting with a pGEX-5X-3-ScPus4 backbone plasmid expressing GST-Pus4 with a Factor Xa cleavage site between the GST tag and Pus4 WT protein sequence (PDG344), the Pus4 R286K was generated using QuikChange Site-Directed Mutagenesis (Agilent Technologies) (**Supp. Table 1**). GST-tagged Pus4 WT and R286K vectors were transformed into *E. coli* BL21(DE3) competent cells (ThermoFisher Scientific). A single transformant of each was inoculated to make a 50 mL overnight starter culture, which was then diluted into a 4 L culture grown until reaching $OD_{600}$=0.80. Cultures were grown at 37°C in TB medium containing 100 μg/mL ampicillin and shaken at 225 rpm. Upon reaching the desired $OD_{600}$, the 4 L culture was shifted to 16°C for 30 minutes. Protein expression was induced by adding IPTG (Gold Bio) to a final concentration of 1 mM, and cells were grown overnight for ~16 hours at 16°C with continuous shaking at 225 rpm. Cells were harvested by centrifugation, and the cell pellets were resuspended in 15 mL of Buffer A (50 mM Tris-HCl pH 7.5, 400 mM NaCl, 10 mM $MgCl_2$, 10% Glycerol, 0.5 mM EDTA, 5 mM BME, 1 tablet of Protease Inhibitor (ThermoFisher Scientific)), divided into two 50 mL conical tubes, and then flash-frozen in liquid nitrogen before storage at -80°C. Each conical contained enough material for a single protein purification. All subsequent procedures were performed at 4°C unless otherwise stated.

Conical tubes were thawed on ice. The following reagents were added to achieve their final concentrations, respectively: lysozyme (Sigma) to 1 mg/mL, DNase (GoldBio) to 10 μg/mL, $MgCl_2$ (Fisher) to 0.5 mM, and RNase A (Fisher) to 30 μg/mL. This cell suspension was incubated for 30 minutes. Cells were lysed via sonication using a sonicator (Branson Sonifier 450) with 30-seconds-on/30-seconds-off cycles (50% duty cycle, power level of 4), repeated three times. Insoluble material was removed by centrifugation for 30 minutes at 30,000 x g in a JA-20 rotor. After clarification, the remaining soluble material was incubated with 2 mL of a 50% slurry of Glutathione-Sepharose beads (Cytiva) that had been pre-equilibrated with Buffer A for 1 hour with end-over-end rotation at room temperature. After incubation, the protein-bound beads were isolated using centrifugation (5 minutes at 500xg) and then washed with 20 mL of Buffer A. This wash step was repeated for a total of five times. Protein was then eluted from the beads with 1 mL of freshly prepared Elution buffer (50 mM Tris-HCl pH 7.6, 10 mM reduced glutathione) after incubating for 5 minutes at room temperature with end-over-end rotation. This elution step was repeated five times and the elutions were pooled together (~5 mL).

The eluate was concentrated to ~1 mL final volume using a 30 kDa Vivaspin concentrator (GE Healthcare). Using the same concentrator, the sample underwent exchange into Buffer B (50 mM Tris-HCl pH 7.5, 100 mM KCl) by adding 10 mL of Buffer B, and performing centrifugation to concentrate to ~1 mL final volume. This was repeated two more times. These protein samples were aliquoted into Eppendorf tubes and flash frozen in liquid nitrogen before storage at -80°C. The yield from each of the two vectors was comparable, and quantified using $A_{280}$ measurements on a Nanodrop spectrophotometer. Protein purity was determined using SDS-PAGE with samples collected across steps of the protein purification process (**Supp. Fig. 1a**).

**Testing purified protein for RNase contamination**

Approximately 1 μg of tRNA isolated from yeast was incubated with 0.4 μM of Pus4 (WT or R286K) in a 10 μL total volume for one hour at room temperature. Control samples of tRNA without protein and tRNA plus protein without incubation were also performed. Samples were combined with 10 μL of RNA loading dye, heated at 70°C for 8 minutes on a heat block, and then loaded onto a fresh pre-run PAGE gel, along with RNA ladder, using an Apogee V16 gel system. After running the gel at 60 mA for about 1.5 hours, the gel was stained with SYBR Gold for 10 minutes at room temperature, and imaged using an Amersham Typhoon (GE) to assess tRNA degradation (**Supp. Fig. 1b**).

**Yeast growth conditions**

*pus4*Δ (YDG127) cells were streaked out onto YPD agar media plates from a frozen -80°C stock and grown at 30°C for 2 days (**Supp. Table 1**). Colonies were inoculated into 5 mL YPD media in culture tubes and grown at 30°C in a rolling drum overnight. In the morning, 400 µL of each culture was transferred to culture tubes with 5.2 mL liquid YPD and grown at 30°C in a rolling drum to an $OD_{600}$ of ~0.8. Cultures were transferred to 15 mL conical tubes and centrifuged at 4°C, 3000xg for 2 minutes. Media was decanted, and the cell pellets were resuspended in 1 mL cold 1X PBS and transferred to microcentrifuge tubes. Tubes were centrifuged at 4°C, 9500 rpm for 1 minute to pellet. Supernatant was decanted and cell pellets frozen by placing microcentrifuge tubes in liquid nitrogen. Cell pellets were stored at -80°C until used for total RNA isolation.

**RNA isolation from yeast**

*pus4*Δ (YDG127) yeast pellets were thawed on ice (**Supp. Table 1**). For cell lysis and RNA preservation, pellets were resuspended in 400 µL TES buffer (10 mM Tris, 10 mM EDTA, 20% SDS). 400 µL acidic phenol was added to each tube. Tubes were vortexed for 10 seconds and incubated in a 65°C water bath for one hour, with 10 seconds of vortexing every 15 minutes. Tubes were put on ice for 5 minutes then centrifuged at 4°C, 13,000 rpm for 10 minutes. The top aqueous layer of each sample was transferred to a new tube. 400 µL of chloroform was added to each of the new tubes. Tubes were vortexed for 10 seconds then centrifuged at 4°C, 13,000 rpm for 10 minutes. The top aqueous layer of each sample was transferred to a new tube and the addition of chloroform, vortexing, and centrifuging repeated. The top aqueous layer of each sample was transferred to a new tube. To precipitate RNA, 1/10 volume of 3 M sodium acetate pH 5.5 and 2.5 volumes of ice cold 100% EtOH was added to each sample and mixed. Tubes were stored at -80°C for one hour. Tubes were centrifuged at 4°C, 13,000 rpm for 10 minutes. The supernatant was removed and 500 µL cold 70% EtOH added to each tube to wash pellets of salt. Tubes were centrifuged at 4°C, 13,000 rpm for 10 minutes. Ethanol was removed and tubes left open for 5-10 minutes to allow residual ethanol to evaporate. Dried pellets were resuspended in 25 µL water, and their concentrations were quantified using a Nanodrop.

An 8% urea acrylamide gel was made using the National Diagnostics UreaGel 19:1 Denaturing Gel System. Gel was pre-run in 1X TBE running buffer at 45 mA for 30 minutes. Total RNA samples were aliquoted to 10 µL volumes and 10 µL 2X RNA loading dye (NEB) added to each sample. 3 µL Low Range ssRNA Ladder (NEB) was mixed with 7 µL water and 10 µL 2X RNA loading dye. Samples were incubated at 70°C for 8 minutes. Samples were loaded on the gel and empty wells loaded with 10 µL water + 10 µL 2X RNA loading dye. Gel was run at 20 mA for 15 minutes and then at 60 mA for ~1 hour. Gel was stained with SYBR gold (Invitrogen) in 1X TBE running buffer for 10 minutes. Gel was imaged with an Amersham Typhoon (GE). Using UV shadowing, tRNA bands were cut from the gel. Gel fragments were placed in tubes with 450 µL 0.3 M NaCl. Tubes were put on a tube inverter overnight at 4°C. Liquid was transferred to new tubes and 1.05 mL 100% EtOH was added to each sample and mixed. Samples were stored at -80°C for one hour. Samples were centrifuged at 4°C, 13,000 rpm for 35 minutes. The supernatant was removed and tubes left open for 5-10 minutes to allow residual ethanol to evaporate. Dried pellets were resuspended in 10-20 µL water and stored at -80°C.

***in vitro* modification**

To perform the *in vitro* modification, 1 µM Pus4 protein was mixed with 300 nM total tRNA harvested from *pus4*Δ cells and incubated at 30°C for 2 minutes in a 200 µL reaction volume in Buffer B (50 mM Tris-HCl pH 7.5, 100 mM KCl). Buffer B supplemented with 4 mM $MgCl_2$ was tested to observe whether magnesium affected catalysis. Since it did not have a strong effect (**Supp. Fig. 2**), $MgCl_2$ was excluded from the buffer used for all smFRET and BLI assays. These reactions were halted with the addition of 200 µL of saturated phenol (pH 6.6). Samples were mixed and then stored at -80°C.

To purify *in vitro* modified tRNA, samples were thawed on ice, then vortexed for 10 seconds and centrifuged at 4°C, 13,000 rpm for 10 minutes. tRNA was re-purified as done in the total RNA isolation, beginning with the addition of 400 µL chloroform. Samples were resuspended in 10 µL water and concentrations quantified using a Nanodrop. Samples were stored at -80°C.

**Direct sequencing of *in vitro* modified tRNA using Oxford Nanopore Technology (ONT)**

To perform library preparation for sequencing, samples were first thawed on ice. Samples were incubated at 95°C for 2 minutes, room temperature for 2 minutes, then on ice for 2 minutes. In a new tube, 7.25 µL tRNA sample was mixed with 2 µL 10X RNA ligase 2 buffer (NEB), 0.8 µL each of: 10 µM ACCA, 10 µM GCCA, 10 µM UCCA, 5 µM CCCA, and 5 µM CCA specific adapters[6], 2 µL 50% PEG, 2 µL 250 mM $MgCl_2$, 1.25 µL 100 mM DTT, 2µL 20 mM ATP, and 1 µL RNA ligase 2 (NEB), for a total volume of 20 µL. The reaction was incubated at room temperature for 45 minutes. 20 µL water was added to the reaction. tRNA was purified with 1.8X volume RNAClean XP Beads (Beckman Coulter). Samples were washed twice with 500 µL 80% EtOH. Samples were eluted with 23 µL water. Samples were mixed with 8 µL 5X quick ligase buffer (NEB), 6 µL RMX adapter (ONT), and 3 µL T4 DNA ligase (2,000,000 U/mL; NEB) for a total volume of 40 µL. Reaction was incubated at room temperature for 30 minutes. tRNA was purified with 1.5X volume RNAClean XP Beads. Samples were washed twice with 150 µL WSB (ONT). Samples were eluted with 15 µL ELB (ONT). Water was added for a total volume of 37.5 µL. Samples were sequenced with MinION devices following the ONT SQK-RNA002 protocol.

Pseudouridylation of $U_{55}$ in tRNA leads to a base miscall, due to an alteration in the ionic current when a pseudouridine transitions through the nanopore, as compared to the different disruption in ionic current caused by the unmodified uridine base. This causes a "mismatch" between the sequenced base and the unmodified reference base. The "reference match probability" refers to the probability that a given base will match the reference base. Therefore, a low reference match probability at position 55 is indicative of a pseudouridylated base. For more details see reference[6].

Sequencing results were basecalled using Guppy v3.0.3, aligned with BWA-MEM (-W 13 –k 6 –xont2d), and quality controlled with SAMtools (Q1). marginAlign was used to create alignment and error models, and marginCaller was used to calculate mismatch probabilities. Python was used to calculate the reference match probability—equal to a value of 1.0 minus the mismatch probability value—and generate heatmaps (**Supp. Fig. 2**). DRS data files are listed in **Supp. Table 3**.

#### RNA preparation for BLI and smFRET

The RNA was diluted in Buffer B to a concentration of 10 nM for smFRET and 60 nM for BLI measurements. The solutions were heated to 90°C on a heat block for 2 minutes, then allowed to cool for 10 minutes at room temperature before placing on ice. The same RNA substrates were used for both BLI and smFRET measurements. For smFRET measurements, the RNA was further diluted to 10 pM with the addition of 5 mM D-glucose (Sigma Aldrich), 165 U/mL glucose oxidase from Aspergillus niger (Sigma Aldrich), 2150 U/mL catalase from Corynebacterium glutamicum (Sigma Aldrich) and 5 mM trolox (Sigma Aldrich) as an oxygen scavenger. This oxygen scavenger was used for RNA slide injection due to its fast acting capability, allowing for a higher level of control over RNA concentration during slide immobilization.

#### Electrophoretic Mobility Shift Assay

The RNA was diluted in Buffer B to 30nM and was incubated with varying concentrations (0, 15 nM, 30 nM, 60 nM, 120 nM, 200 nM, 400 nM, 600 nM, 800 nM) of Pus4 in a total volume of 10 μL for 30 minutes at room temperature. Each 10 μL solution was mixed with 10 μL of loading buffer (2x TBE, 40% glycerol). The mixtures were run on an 8% Polyacrylamide native/non-denaturing gel at 300V for 2.5 hours at 4ºC. The gel was prepared from 40% 19:1 Acrylamide/Bisacrylamide (Bio-Rad), TEMED (Thermo Fisher Scientific), and Ammonium Persulfate (Thermo Fisher Scientific) and pre-run in 1X TBE running buffer at 300 V for 90 minutes. The gel was imaged using an Amersham Typhoon (GE).

#### Biolayer Interferometry (BLI)

BLI measurements were performed on a GatorPlus Biolayer Interferometer (GatorBio, Palo Alto, CA) utilizing proprietary GatorOne software for data collection. All BLI measurements were obtained at 30°C. GatorBio streptavidin-coated glass probes were presoaked in Buffer B. Probes were presoaked in Buffer B for at least 20 minutes before collecting baseline signal for 300 seconds. The probes were immersed in 60 nM biotinylated tRNA until an adequate shift (~1.2 nm) in signal was observed (~60 seconds). The probes were then submerged in 1:1000 biocytin in Buffer B to quench the remaining streptavidin sites. This was followed by a second quenching in Buffer B supplemented with 0.2% Tween-20 in (“Buffer C”), which also aided in reducing non-specific protein binding. For this reason, Buffer C was used for all following steps. Loaded probes were then incubated in Buffer B until baseline was achieved (~300 seconds). To measure association of the protein to the tRNA, the loaded probes were then incubated in 200 nM of GST-Pus4 (WT or R286K) in Buffer C for ~3000 seconds. To measure dissociation, the probes were then submerged in Buffer C for the same amount of time as the association step. Reference probes were included in some runs to assess non-specific protein binding and RNA dissociation from the probe.

#### Analysis of BLI data

To estimate the kinetics of WT Pus4 binding to tRNA, we attempted to isolate the initial rate of association before pseudouridylation (in the case of tRNA that was not premodified) or refolding occurred. In isolation, this early phase of binding represents a reversible second order reaction

(RNA+Pus4 ⇋ RNA·Pus4). We approximated its kinetics as pseudo-first order in Pus4, as the protein was present in bulk solution while the quantity of tRNA was limited by the number of binding sites on the probe. This is expected to yield an exponential shape to the association curve (**Eq. 1**):

(1) $$[RNA \cdot Pus4]_t = [RNA]_0(1 - e^{-k_{obs}t})$$

where the observed rate of the reaction is:

$$k_{obs} = k_{on}[Pus4] - k_{off}$$

Considering that at each timepoint *t*, $[RNA]_0 = [RNA \cdot Pus4]_t + [RNA]_t$, and that at the final timepoint $[RNA]_{final} = [RNA]_0 - [RNA \cdot Pus4]_{final}$, **Eq. 1** becomes:

(2) $$\frac{[RNA \cdot Pus4]_{final} - [RNA \cdot Pus4]_t}{[RNA \cdot Pus4]_{final}} = e^{-k_{obs}t}$$

To estimate $k_{on}$ and $k_{off}$, the association phases of the BLI curves were converted to fractional saturation (FS) using **Eq. 3**, where *s(t)* is the shift at time *t* and $s_{final}$ is the shift at the final timepoint of the association phase:

(3) $$FS(t) = 1 - \frac{s_{final} - s(t)}{s_{final}}$$

Two methods were then used to fit the fractional saturation curve: 1) The early segment of the association phase over which ~75% of the overall shift occurred was fitted with a single exponential (**Eq. 4**), or 2) the entire association phase was fitted with the sum of 2 exponentials (**Eq. 5**). In **Eq. 5**, the faster of the two rates was taken to be $k_{obs}$.

(4) $$FS(t) = y - a \cdot e^{-k_{obs}t}$$

(5) $$FS(t) = y - a_1 \cdot e^{-k_1 t} - a_2 \cdot e^{-k_2 t}$$

These fits were performed for BLI curves obtained at 50 nM, 200 nM and 800 nM Pus4. $k_{obs}$ was then plotted against [Pus4] and the slope of a linear fit yielded an estimate for $k_{on}$. In principle, the y-intercept of the fit yields $k_{off}$. However, determining it accurately would require measurements at lower [Pus4], conditions that reduce both the accuracy of the pseudo-first order kinetics approximation and the separation of the timescales on which Pus4 accumulates on the probe and on which it modifies and/or remodels the tRNA it has already bound to. Because $k_{obs}$ was less than 0.01 $s^{-1}$ at 50 nM Pus4 (the lowest concentration tested) for all RNAs and fitting approaches, we can reasonably assume that $k_{off}$ is less than that value. Given the values obtained for $k_{on}$ (**Supp. Fig. 6**), this constrains the dissociation constant $K_D = k_{off}/k_{on}$ to a value less than ~130 nM.

**Single molecule FRET (smFRET)**

The RNA of interest, prepared as described above, was flowed onto a slide that had been passivated with DDS (Sigma Aldrich), incubated with 0.2 mg/mL BSA (Thermo Fisher Scientific) for 5 minutes, 0.2% v/v Tween-20 for 10 minutes, and 0.2 mg/mL streptavidin (Thermo Fisher Scientific) for 5 minutes. Imaging was performed with Buffer B containing 5 mM 3,4-dihydroxybenzoic acid (Sigma Aldrich), 5 mM Trolox (Sigma Aldrich), and 50 nM Protocatechuate 3,4-dioxygenase (Sigma Aldrich) as an oxygen scavenging system. 200 nM Pus4 WT or R286K in Buffer C containing the above oxygen scavenger was prepared and allowed to tube incubate with minimal headspace for 10 minutes before injection into the system. This pre-incubation allowed for movie capture as early as two minutes after injection. Data were collected on a home-built mtprism-type total internal reflection fluorescence (TIRF) microscope (Leica DMi8 inverted microscope, 63x oil immersion objective). Cy3 and Cy5 were excited by a 532 nm laser (Coherent OBIS 532 LS) at 140 mW and a 637 nm laser (Coherent OBIS 637 LX) at 60 mW, respectively. The slide was illuminated at 532 nm continuously and at 637 nm at the beginning and end of each movie to confirm that Cy5 was active. tRNA movies were recorded on an Andor iXon 888 EMCCD with an exposure time of 100 ms. Two minute movies were collected back to back for the first 15 minutes following protein injection. Additional movies were collected 30 minutes after protein injection.

Coordinates for each molecule were extracted from raw video files using FIJI and fluorophore intensity traces were extracted and corrected for crosstalk and detector sensitivity using custom MATLAB code. Traces that exhibited single-step photobleaching of Cy3 and Cy5 and anticorrelation between donor and acceptor signals when transitions occurred were selected manually and analyzed using custom MATLAB codes. The FRET efficiency was calculated as $I_A/(I_A+I_D)$, where $I_A$ and $I_D$ are the background corrected fluorescence intensities of Cy5 and Cy3 respectively. FRET histograms were compiled from the first 50 frames of each selected trace for a large number of traces (>200) and fitted with a sum of three Gaussian functions in Mathematica (Wolfram Alpha). Hidden Markov Modeling was performed in QuB (Quantifying unknown Biophysics) Online[58–60] and TODPs were generated from the resulting idealized traces using custom MATLAB codes. Cross-correlation analysis was performed in Mathematica by computing the cross-correlation C(τ) between Cy3 and Cy5 intensities using Eq. 6.

$$C(\tau) = \frac{\langle \Delta Cy3(t)\Delta Cy5(t+\tau)\rangle}{\langle \Delta Cy3(0)\Delta Cy3(t)\rangle^{1/2}\langle \Delta Cy5(0)\Delta Cy5(t)\rangle^{1/2}} \qquad (6)$$

where $\Delta X(t) = X(t) - \langle X \rangle$ and $\langle X \rangle$ is the average value of X across all frames t within the trace.

**Free energy calculations**

The free energies of different secondary structures were predicted using the RNAfold web server[61]. The sequence used for mt-tRNA$^{Lys}$ was the one utilized in Voigts-Hoffman *et al.*, 2007 with U instead of dT at its fluorophore labeling sites, and the sequence used for tRNA$^{eMet(CAU)}$ was the one utilized in this work. Mild base-pairing constraints were applied to achieve the C

and E secondary structures of mt-tRNA$^{Lys}$ as depicted in Fig. 1 of Voigts-Hoffman *et al*., 2007 (1 and 2 enforced BP, respectively), the canonical tRNA$^{eMet(CAU)}$ structure as depicted in **Fig. 1** of this work (2 enforced BP), and a plausible E-like structure of tRNA$^{eMet(CAU)}$ (4 enforced BP). The secondary structures were optimized under these constraints with energy parameters re-scaled to 25°C and 0.1 M salt and isolated BP allowed. The resulting minimum free energy structures were re-rendered using R2DT[62] to generate the visualizations shown in **Supp. Fig. 10**.

### Data Availability

The direct RNA sequencing data is publicly accessible at the European Nucleotide Archive https://www.ebi.ac.uk/ena (accession number PRJEB85651).

### Acknowledgments

We thank Ute Kothe (University of Manitoba) for the generous gift of a Pus4 expression plasmid that we modified for use in this study. We thank Laura McKnight (Knight Campus for Accelerating Scientific Impact) for guidance on protein purification. We thank Marian Hettiaratchi, Parisa Hosseinzadeh and Justin Svendsen (Knight Campus for Accelerating Scientific Impact) for the use and training of their biolayer interferometry instrument. We thank Alice Barkan, Brad Nolen and Preeti Bhattacharjee (University of Oregon) for their comments on the manuscript.

### Funding

This work was supported by the National Institutes of Health (R35 GM147229 to J.R.W., R35 GM143125 and R01 HG013876 to D.M.G., and T32 GM007413 to E.M.D.) and by the University of Oregon Center for Optical, Molecular and Quantum Science (Emanuel Fellowship to N.E.C.N.).

### Contributions

D.M.G. and J.R.W. conceived the project. E.M.D., N.E.C.N., A.L.V., M.K., M.L.B., D.M.G. and J.R.W. conducted the investigation and performed the formal analysis, data curation and visualization. All authors contributed to the writing and editing of the manuscript.

### Ethics Declarations

The authors declare no competing interests.

## Supplementary Files

This is a list of supplementary files associated with this preprint. Click to download.

- DennisXConoanNieves2025MainTextFINAL.pdf
